# Effect of Glucagon-like Peptide-1 Receptor Agonists on Outcomes After Hip Hemiarthroplasty for Femoral Neck Fractures in Patients With Type 2 Diabetes

**DOI:** 10.5435/JAAOSGlobal-D-25-00312

**Published:** 2025-10-17

**Authors:** McKenna W. Box, Troy B. Puga, Neil J. Werthmann, Yingxian Liu, John T. Riehl

**Affiliations:** From the Department of Orthopaedic Surgery, Medical City Denton, Denton, TX (Dr. Box, Dr. Puga, Dr. Werthmann, and Dr. Riehl), and the Physician Services Group, Graduate Medical Education, HCA Healthcare, Brentwood, TN (Ms. Liu).

## Abstract

The use of glucagon-like peptide-1 receptor agonists (GLP-1RA) in patients with type 2 diabetes mellitus (T2DM) has increased substantially over the past several years. The purpose of this study was to determine whether GLP-1RA use affects outcomes after hip hemiarthroplasty (HA) for femoral neck fractures (FNFs). A retrospective cross-sectional analysis of a local hospital system database was conducted between 2016 and 2023 to identify patients with T2DM aged at least 18 years who underwent HA for FNFs and were on a GLP-1RA at the time of injury. A 1:1 random patient sample of those who underwent HA and were not on a GLP-IRA was used as a control. Patient characteristics and Elixhauser comorbidity index were recorded. Outcomes included hospital length of stay, aspiration pneumonitis during index hospitalization, inpatient readmissions and emergency department encounters within 365 days, medical complications, surgical site infection, implant complications, revision hip surgery, and in-hospital mortality/discharge to hospice within 30, 90, and 365 days. Binary logistic regression was done to assess the 30-day risk of medical and the 365-day risk of implant postoperative outcomes. Four hundred ninety-nine patients (GLP-1RA, N = 248; No GLP-1RA, N = 251), with T2DM, who underwent HA for FNF were included for analysis. GLP-1RA use was not markedly associated with medical complications within 30, 90, or 365 days; implant complications or revision surgery within 365 days; in-hospital mortality/discharge to hospice within 30 or 90 days; postoperative aspiration; length of stay; or inpatient readmissions or emergency department encounters. GLP-IRA use was associated with a decreased risk of in-hospital mortality/discharge to hospice within 365 days. When controlling for confounding variables, the use of GLP-IRA was not associated with any adverse outcome measured in the study (*P* > 0.05). GLP-1RA use in T2DM patients undergoing HA for FNF is not associated with an increased risk of early postoperative medical and surgical complications.

Hip hemiarthroplasty (HA) remains one of the most common procedures in the world for treating displaced femoral neck fractures (FNF).^1^ With an aging population, the incidence of FNFs and HA will likely continue to rise over the next decade.^2^ FNFs are noteworthy injuries in the elderly population because they have a notable 30-day and 1-year mortality rate.^3^ Urgent treatment within 48 hours has been shown to reduce the 30-day mortality; however, there remains a roughly 30% mortality rate at 1 year.^3,4^ HA has several well-studied complications such as infection, dislocation, periprosthetic fractures, and aseptic loosening.^4^

In addition to an aging population, the rates of obesity, type 2 diabetes mellitus (T2DM), and cardiovascular disease are rapidly continuing to climb.^5,6^ T2DM and obesity have been associated with decreased femoral neck bone density, increasing the risk of fracture.^7,8^ In addition, T2DM and obesity are a notable concern in arthroplasty surgery because they are associated with an increased risk of complications, particularly infections and revision surgeries.^9-11^ Over the past several years, notable improvements have been made in treating obesity and T2DM.^12^ One example is the recent increase in usage of glucagon-like peptide-1 receptor agonists (GLP-1RA) in the medical treatment of T2DM and obesity.^13,14^

GLP-1RAs (such as semaglutide) are a class of synthetic medications effective at improving glycemic control for T2DM and have been increasingly used as weight loss therapy over the past several years.^13,14^ GLP-1RA's mimic the effects of GLP-1 in the body by increasing glucose-dependent insulin secretion, reducing glucagon secretion, decreasing appetite, and decreasing the central drive for food cravings.^15^ These medications have shown high levels of efficacy in glycemic control and weight loss through numerous clinical trials.^16-19^

Although there has been literature evaluating the outcomes of GLP-1RA use in elective total hip arthroplasty (THA)^20-23^ and total knee arthroplasty,^20,24,25^ the literature remains scant, particularly in the trauma setting. To our knowledge, this is the first study to evaluate the outcomes of GLP-1RA medications in HA for FNFs in diabetic patients. This study aims to determine whether the use of GLP-1RA in patients with T2DM affects outcomes after HA for FNF. We hypothesize that GLP-1RA medications will reduce the overall complications and improve the outcomes for diabetics undergoing HA for FNF.

## Methods

### Study Design and Setting

A retrospective cross-sectional study of prospectively collected data was done using deidentified electronic health record data from eight community hospitals in North Texas that belong to a large healthcare system in the United States. The Institutional Review Board approved this study as exempt under category 4 (secondary analysis of existing data sets). The study findings are per the Strengthening the Reporting of Observational Studies in Epidemiology guidelines.

### Participants

Inclusion criteria for this study were adults aged 18 years and older, with diagnosis of T2DM admitted from the community to acute inpatient units from January 1, 2016, to August 28, 2023, who received International Classification of Disease, 10th Revision, Clinical Modification (ICD-10) diagnoses of FNF (S72.0), and had Current Procedural Terminology (CPT) codes for HA (27125 or 27236). Because CPT 27236 includes internal fixation of FNF fractures, the presence of ICD-10-Procedure Coding System codes for “Replacement of Hip Joint, Femoral Surface” (0SRS, 0SRR) was also necessary to ensure only HA was included. Patients admitted for revision hip surgery, those treated with THA, patients who left against medical advice, patients without demographic variables reported, and patients with a history of rheumatoid arthritis or concurrent myocardial infarction, traumatic brain injury, or other fractures were excluded. Overall, 291 primary encounters of HA to treat a FNF in patients with T2DM taking a GLP-1RA at the time of surgery were reported.

The comparator group of patients with T2DM not taking a GLP-1RA with a FNF treated with HA was selected through 1:1 random sampling from the same hospital system during the study period. No specific matching algorithm or time-based matching was done. Although patients were not explicitly matched by year of surgery, subsequent multivariable regression analyses were conducted, adjusting for key covariates (age, sex, body mass index [BMI] class, smoking status, and Elixhauser Comorbidity Index [ECI]) to minimize confounding and address baseline differences between groups.

### Variables and Outcome Measures

Demographic variables included age, sex, smoking status, race, BMI, and the ECI for medical comorbidities.^26,27^ ECI scores are based on the dichotomous presence of 31 comorbidities using ICD diagnosis codes. ECI accurately predicts readmissions and mortality in total joint arthroplasty patients.^28-30^ Admission hemoglobin A1c (HgbA1c) was recorded when available.

Where available, ICD-10-Procedure Coding System procedure codes were used to determine the HA fixation technique (cemented [0SR*0*9], press-fit [0SR*0*A], or no qualifier [0SR*0*Z]). Specific details regarding the surgical approach (eg, anterior, anterolateral, and posterior) and individual surgeon information were unavailable and therefore not included in the analysis.

The variables analyzed included medical complications (cerebrovascular accident, deep vein thrombosis, pulmonary embolism, myocardial infarction, pneumonia, acute kidney injury, and hypoglycemic event) and surgical site infection (SSI) within the first 30 days, 90 days, and 1 year. The incidence of aspiration pneumonitis during the index hospitalization was evaluated using ICD-10-Clinical Modification diagnostic codes (J69.0). Reencounters were evaluated as inpatient readmissions (IRs) or emergency department encounters (EDEs) within a 1-year period. Implant complications analyzed included periprosthetic fracture, instability, prosthetic joint infection (PJI), aseptic loosening, other implant complications (broken internal prosthesis, dislocation, periprosthetic osteolysis, wear of the articular bearing surface of the internal prosthesis, and other mechanical complications), and revision hip arthroplasty within 1 year after HA. Individual complications were counted separately, but IRs or EDE was uniquely counted, regardless of the presence of multiple concurrent diagnoses.

### Statistical Analysis

The demographic and clinical characteristics of the patient sample were described using descriptive statistics, using means and SD for continuous variables and frequencies and percentages for categorical variables. Comparisons across groups were conducted using the Pearson chi-square test for categorical outcomes with adequate expected counts and the Fisher exact test for those with small expected counts. The Wilcoxon rank sum test was used for the pairwise comparison of continuous variables. Bonferroni correction was applied to control for the familywise error rate for multiplicity. Effect sizes were determined using the rank-biserial correlation, *r*, for continuous variables, and Cramér V, for categorical variables.

Binary logistic regression models were used to examine the association between GLP-1RA use and outcomes while adjusting for diabetes status, age, sex, BMI group, smoking status, and ECI. Firth correction was applied for rare outcomes, including 1-year implant complications, revision surgery within 1 year, and 30-day mortality. Standard binary logistic regression was used for IRs or EDEs and 30-day medical complications. Adjusting for the same covariates, negative binomial regression was used to assess the association between GLP-1RA use and hospital length of stay (LOS). A *P*-value of < 0.05 was considered statistically significant. All analyses were conducted using SAS software, version 9.4 (SAS Institute).

## Results

### Participants and Descriptive Data

Two hundred ninety-one patients with T2DM on a GLP1-RA who underwent HA for FNF were identified. Three hundred patients with T2DM who were not on a GLP-1 RA and underwent HA for FNF were randomly selected as comparators. After applying the exclusion criteria, 499 patients (GLP-1RA, N = 248; No GLP-1RA, N = 251) remained for analysis.

Of patients with T2DM who underwent HA for FNF, the GLP-1RA group was significantly younger (mean age 74.9 ± 7.6 versus 78.2 ± 9.4 years, *P* < 0.001) and had a higher mean BMI (28.9 ± 5.9 versus 26.1 ± 5.8 kg/m^2^, *P* < 0.001) compared with those not on GLP-1RA (Table 1). No differences were noted in sex distribution (*P* = 0.530) or race group (*P* = 0.166). Both cohorts had similar comorbidity burdens (ECI 4.5 ± 1.9 versus 4.5 ± 1.9, *P* = 0.755), and there were no significant differences in length of hospital stay (*P* = 0.072) or time from injury to surgery (*P* = 0.945). However, patients on GLP-1RA had higher admission HbA1c levels (8.0 ± 2.1 versus 7.1 ± 1.7, *P* < 0.001). In addition, there was no significant difference in the rate of aspiration pneumonitis between GLP-1RA users and nonusers during the index admission (*P* = 0.750).

**Table 1:** Demographics of Patients With and Without GLP-1 Receptor Agonist Use Undergoing Hemiarthroplasty for Femoral Neck Fractures

Variable^a^	GLP-1RA N = 248	No GLP-1RA N = 251	*P* ^b^	Effect Size^c^
Age (yr)				
Overall	74.9 (7.6)	78.2 (9.4)	**<0.001**	0.221
Age group			**<0.001**	0.028
41-60	9 (3.6%)	12 (4.8%)		
61-80	185 (75%)	127 (51%)		
81+	54 (22%)	112 (45%)		
Sex			0.530	0.028
Female	155 (63%)	150 (60%)		
Male	93 (38%)	101 (40%)		
Race			0.393	0.091
White	110 (44%)	96 (38%)		
African American	122 (49%)	134 (53%)		
Asian	2 (0.8%)	5 (2.0%)		
Hispanic	1 (0.4%)	0 (0%)		
Other	13 (5.2%)	16 (6.4%)		
BMI (kg/m^2^)				
Overall	28.9 (5.9)	26.1 (6.0)	**<0.001**	0.273
BMI class			**<0.001**	0.283
Underweight (<18.5)	3 (1.2%)	14 (5.6%)		
Normal (18.5-24.9)	45 (18%)	89 (35%)		
Overweight (25-29.9)	108 (44%)	99 (39%)		
Class 1 obesity (30-34.9)	55 (22%)	38 (15%)		
Class 2 obesity (35-39.9)	24 (9.7%)	5 (2.0%)		
Class 3 obesity (>40)	13 (5.2%)	6 (2.4%)		
ECI	4.5 (1.9)	4.5 (1.9)	0.755	0.014
ESRD	9 (3.6%)	20 (7.9%)	0.380	—
Admission Hgb A1c (%)	8.0 (2.1) N = 99	7.1 (1.7) N = 102	**<0.001**	0.239
Current smoker	24 (9.7%)	22 (8.8%)	0.725	0.016
Days from injury to surgery (d)	1.2 (1.3)	1.1 (1.2)	0.945	0.003
LOS (d)	5.9 (4.0)	6.3 (3.7)	0.072	0.081
HA fixation method			0.114	0.104
Cemented	61 (25%)	44 (18%)		
Press fit	48 (19%)	60 (24%)		
Not specified	139 (56%)	147 (59%)		

BMI = body mass index, ECI = Elixhauser comorbidity index, ESRD = end-stage renal disease, GLP-1RA = glucagon-like peptide-1 receptor agonist, HA, hemiarthroplasty, LOS = length of stay

a Mean and SD for continuous variables. Frequency (N) and percentage for categorical variables.

b Chi-squared test or Fisher exact test for categorical variables and the Wilcoxon rank sum test for continuous variables. *P* values adjusted for multiple comparisons through the Bonferroni correction. Values in bold indicate statistical significance (*P* < 0.05).

c Rank-biserial correlation, r, for continuous variables and Cramér, V, for categorical variables equal to 0.10 to 0.3 reflects a small effect, 0.30 to 0.50 a medium effect, and >0.50 a large effect.

### Medical Complications

At 30 days postoperatively, rates of acute kidney injury (18% versus 20%, *P* = 0.614), hypoglycemic events (2.4% versus 2.8%, *P* = 0.796), and other measured complications did not differ significantly between the GLP-1RA and non–GLP-1RA groups (Table 2). Similar findings were observed at 90 days, with no significant differences in cerebrovascular accident (1.6% versus 1.2%, *P* = 0.723), pulmonary embolism (2.4% versus 3.2%, *P* = 0.603), or total medical complications (18% versus 19%, *P* = 0.776). At 1 year, IRs (36% versus 32%, *P* = 0.344) and EDEs (17% versus 14%, *P* = 0.143) were similar, and there was no difference in total medical complications (27% versus 29%, *P* = 0.538). Notably, the GLP-1RA group experienced a lower rate of discharge to hospice or mortality at 1 year (10% versus 16%, *P* = 0.039), although the effect size was negligible (V = 0.092).

**Table 2:** Medical Outcomes of Patients With and Without Glucagon-like Peptide-1 Receptor Agonist Use Undergoing Hemiarthroplasty for Femoral Neck Fractures

Variable^a^	GLP-1RA N = 248	No GLP-1RA N = 251	*P* b	Effect Size^c^
Index hospitalization				
Aspiration pneumonitis	5 (2%)	4 (2.6%)	0.750	0.016
30-d outcomes				
Medical complications				
CVA	2 (0.8%)	1 (0.4%)	0.622	0.026
PE	4 (1.6%)	4 (1.6%)	>0.999	—
PNA	0 (0%)	1 (0.4%)	>0.999	—
AKI	25 (10%)	24 (9.6%)	0.846	0.009
Hypoglycemic event	6 (2.4%)	7 (2.8%)	0.796	0.012
Total	31 (13%)	34 (14%)	0.729	0.016
Mortality	10 (4.0%)	15 (6.0%)	0.320	0.045
90-d outcomes				
Medical complications				
CVA	4 (1.6%)	3 (1.2%)	0.723	0.018
DVT	1 (0.4%)	1 (0.4%)	>0.999	—
PE	6 (2.4%)	8 (3.2%)	0.603	0.023
PNA	0 (0%)	1 (0.4%)	>0.999	—
AKI	30 (12%)	34 (14%)	0.557	0.026
Hypoglycemic event	8 (3.2%)	12 (4.8%)	0.376	0.04
Total	44 (18%)	47 (19%)	0.776	0.013
Mortality	17 (6.9%)	21 (8.4%)	0.524	0.028
1-yr outcomes				
Acute inpatient readmissions	75 (30.2%)	70 (27.9%)	0.344	0.042
Emergency department visits	42 (16.9%)	36 (14.3%)	0.143	0.080
Medical complications				
CVA	8 (3.2%)	6 (2.4%)	0.572	0.025
DVT	1 (0.4%)	1 (0.4%)	>0.999	—
PE	13 (5.2%)	14 (5.6%)	0.868	0.007
PNA	0 (0%)	4 (1.6%)	0.124	0.089
AKI	49 (20%)	49 (20%)	0.947	0.003
Hypoglycemic event	16 (6.5%)	25 (10.0%)	0.154	0.064
Total	66 (27%)	73 (29%)	0.538	0.028
Mortality	25 (10%)	41 (16%)	**0.039**	0.092
Fragility fracture	0 (0%)	2 (0.8%)	0.499	0.063

AKI = acute kidney injury, CVA = cerebrovascular accident, DVT = deep vein thrombosis, GLP-1RA = glucagon-like peptide-1 receptor agonist, PE = pulmonary embolism, PNA = pneumonia

a Mean and SD for continuous variables. Frequency (N) and percentage for categorical variables.

b Chi-squared test or Fisher exact test for categorical variables and the Wilcoxon rank sum test for continuous variables. *P* values adjusted for multiple comparisons through the Bonferroni correction. Values in bold indicate statistical significance (*P* < 0.05).

c Rank-biserial correlation, *r*, for continuous variables and Cramér, V, for categorical variables equal to 0.10 to 0.3 reflects a small effect, 0.30 to 0.50 a medium effect, and >0.50 a large effect.

### Surgical Complications

Surgical complication rates did not significantly differ between the GLP-1RA and non–GLP-1RA cohorts at 30 days, 90 days, or 1 year (Table 3). Specifically, there were no significant differences in SSI, PJI, periprosthetic fracture, aseptic loosening, or all-cause revision surgery across all time points (*P* > 0.05).

**Table 3:** Surgical Outcomes of Patients With and Without Glucagon-like Peptide-1 Receptor Agonist Use Undergoing Hemiarthroplasty for Femoral Neck Fractures

Variable^a^	GLP-1RA N = 248	No GLP-1RA N = 251	*P* ^b^	Effect Size^c^
30-d outcomes				
SSI	2 (0.8%)	4 (1.6%)	0.686	0.036
Implant-related complications				
Aseptic loosening	0 (0%)	1 (0.4%)	>0.999	—
PJI	2 (0.8%)	4 (1.6%)	0.686	0.036
PPFx	0 (0%)	4 (1.6%)	0.124	0.089
Other complications^d^	6 (2.4%)	3 (1.2%)	0.337	0.046
Total	7 (2.8%)	9 (3.6%)	0.629	0.022
Revision surgery	3 (1.2%)	5 (2.0%)	0.724	0.031
90-d outcomes				
SSI	2 (0.8%)	6 (2.4%)	0.285	0.063
Implant-related complications				
Aseptic loosening	0 (0%)	1 (0.4%)	>0.999	—
PJI	3 (1.2%)	6 (2.4%)	0.504	0.044
PPFx	3 (1.2%)	6 (2.4%)	0.504	0.044
Other complications^d^	7 (2.8%)	3 (1.2%)	0.220	0.058
Total	11 (4.4%)	13 (5.2%)	0.698	0.017
Revision surgery	7 (2.8%)	6 (2.4%)	0.762	0.014
1-yr outcomes				
SSI	2 (0.8%)	7 (2.8%)	0.176	0.074
Implant-related complications				
Aseptic loosening	0 (0%)	1 (0.4%)	>0.999	—
PJI	4 (1.6%)	9 (3.6%)	0.167	0.062
PPFx	4 (1.6%)	7 (2.8%)	0.371	0.04
Other complications^d^	9 (3.6%)	7 (2.8%)	0.594	0.024
Total	14 (5.6%)	18 (7.2%)	0.487	0.031
Revision surgery	10 (4.0%)	10 (4.0%)	0.978	0.001

GLP-1RA = glucagon-like peptide-1 receptor agonist, PJI = prosthetic joint infection, PPFx = periprosthetic fracture, SSI = surgical site infection

a Frequency (N) and percentage are presented for categorical variables.

b Chi-squared test or Fisher exact test for categorical variables. *P* values were adjusted for multiple comparisons through the Bonferroni correction. Values in bold indicate statistical significance (*P* < 0.05).

c Rank-biserial correlation, *r*, for continuous variables and Cramér, V, for categorical variables equal to 0.10 to 0.3 reflects a small effect, 0.30 to 0.50 a medium effect, and >0.50 a large effect.

d Broken internal prosthesis, dislocation, periprosthetic osteolysis, wear of articular bearing surface of internal prosthetic, other mechanical complications

### Outcome Regression Analysis

No regressions were conducted for SSI within 30 days (N = 6, 1.2%) and fragility fracture within 1 year (N = 2, 0.4%) due to insufficient cases to support a reliable analysis. Binary logistic regression analyses revealed no statistically significant association between GLP-1RA use and the odds ratio of 30-day medical complications or mortality, nor with 1-year IRs or EDEs, implant complications, all-cause revision surgery, or mortality after adjusting for potential confounders (Table 4). Of the other control variables, ECI was associated with increased LOS, 30-day medical complications, IRs or EDEs, mortality, and increased 1-year mortality. Supplemental Digital Content Table 1, http://links.lww.com/JG9/A457, summarizes the results from the regression analysis for each control variable.

**Table 4:** Regression Analysis of Outcomes of Patients With and Without Glucagon-like Peptide-1 Receptor Agonist Use Undergoing Hemiarthroplasty for Femoral Neck Fractures

Variable	Odds Ratio^a^	95% CI	*P* ^b^
Total LOS (d)^c^	IRR = 0.909	0.825 to 1.002	0.0538
30-d outcomes			
Medical complications	0.907	0.566 to 1.453	0.6844
Mortality/discharge to hospice	0.827	0.343 to 1.996	0.6727
1-yr outcomes			
IRs or EDEs	1.045	0.710 to 1.538	0.824
Total implant complications	0.605	0.291 to 1.256	0.1776
Revision surgery	0.825	0.347 to 1.959	0.6621
Mortality	0.693	0.384 to 1.248	0.2216

95% CI = 95% confidence interval, BMI = body mass index, ECI = Elixhauser comorbidity index, EDEs = emergency department encounters, IRR, IRs = inpatient readmissions, LOS = length of stay, OR = odds ratio

a Controlled for age, sex, ECI, smoking status, and BMI class.

b Statistical significance set at *P* < 0.05.

c Negative binomial regression of the log count of initial hospital length of stay.

## Discussion

In this retrospective cross-sectional study of patients with T2DM undergoing HA for FNF, we observed that patients prescribed GLP-1RAs were younger, had higher BMIs, and presented with higher baseline HbA1c levels compared with a random sample population of T2DM nonusers. Despite these differences, short-term and medium-term outcomes, including 30- and 90-day complications, IRs or EDEs, and mortality, were comparable between groups. Unadjusted analysis suggested that GLP-1RA use was associated with a lower rate of discharge to hospice or mortality at 1 year. However, this association did not reach significance in the adjusted models. These findings indicate that GLP-1RA use is likely not associated with an effect on short-term outcomes after HA for FNF.

To our knowledge, our study is the first to evaluate the effects of GLP-1RA medications in diabetic patients undergoing HA for FNF. By contrast, in some previous investigations in the elective THA population, GLP-1RA use was associated with decreased PJIs and IRs or EDEs.^20,23^ Elevated blood glucose levels are a well-recognized risk factor for postoperative infections, and the glucose-lowering properties of GLP-1RAs may help mitigate this risk.^31^ In addition, given the observed benefit of weight loss with GLP-1RA therapy, these agents might reduce BMI-related complications after HA for FNF.^32^ The dual metabolic and weight-loss effects of GLP-1RAs may offer an advantage in the overall complication profile. Our data suggest that the comorbidity burden, as captured by the ECI, remains a notable determinant of outcomes in this high-risk population.

Several factors may explain the limited effect on short-term complications in our cohort. The observed younger age and higher BMI among GLP-1RA users might indicate a selection bias, where more motivated patients or those with fewer comorbidities are preferentially started on these agents for improved long-term glycemic management. In addition, glycemic control during the perioperative period is multifactorial, shaped by overall T2DM management, nutritional status, and the physiologic stress of surgery. These elements may obscure the isolated effect of GLP-1RA therapy on immediate postoperative results. The anti-inflammatory and cardiometabolic benefits of GLP-1RAs require a longer duration to become clinically evident, thus potentially not influencing 30- or 90-day outcomes as markedly as 1-year measures or longer.

Although HA for FNF is inherently associated with high complication rates, our findings suggest that GLP-1RA use does not exacerbate these risks and might contribute to improved long-term survival. The mortality and complication rates observed in our diabetic cohort were comparable with those reported for the general population, where 1-year mortality ranges from 20% to 30% and early complication rates between 15% and 25%.^3,4^ Ekström et al^33^ showed that 1 year after FNF, diabetic and nondiabetic patients had similar medical complications; however, at the 2-year mark, patients with diabetes had markedly higher medical complications. Whether GLP-1RA use confers an overall net positive or negative effect beyond the 1-year mark after HA for FNF is unclear. Additional longitudinal evaluation should be done to determine whether GLP-1RAs mitigate this risk, which was not evaluated at the time of this study.

In our study, the incidence of aspiration pneumonitis during the index hospitalization did not differ markedly between GLP-1RA users and nonusers, suggesting no substantial association with perioperative aspiration risk in urgent hip fracture surgery. These findings align with recent studies reporting no elevated perioperative aspiration risk associated with GLP-1RA use.^34,35^ However, our study lacked information on the length of GLP-1RA use, anesthesia type, airway management strategies, and preoperative symptomatology suggestive of delayed gastric emptying. These are contextual factors emphasized in the 2024 American Society of Anesthesiologists guidelines,^36^ which suggest continuing GLP-1RA through the day of surgery in low-risk patients. Nonetheless, there is a need for prospective studies to assess the perioperative management of GLP-1RAs, particularly in urgent surgical situations where preoperative medication adjustments are impractical.

### Limitations

The findings from this retrospective cross-sectional analysis should be interpreted within the context of the study's limitations. Our findings are limited to patients undergoing HA for FNF within a single hospital system. These factors may affect the generalizability of the findings to other hip fracture subtypes, surgical modalities, or geographic regions. In addition, complications may have been underreported if patients received care at outside hospitals. Our data relied on ICD-10 and CPT coding, which are susceptible to errors or omissions, potentially underreporting some outcomes.

Temporal variables such as the year and hospital of presentation were not controlled for, which could introduce confounding. However, to avoid overfitting, we prioritized adjustment for clinically relevant covariates. In addition, the cohorts collected span from 2016 to 2023. Since GLP-1RA use has grown markedly since 2023, larger contemporary cohorts may better delineate effects.

Procedural details such as surgical approach, anesthesia type, and surgeon identifiers were unavailable because of database limitations. Although these variables potentially have minimal effect on long-term results, their exclusion hindered our capacity to evaluate procedural variability.^37,38^ Postoperative glucose control measures, timing of GLP-1RA resumption, patients on dialysis, or deep vein thrombosis prophylaxis regimens were unavailable, limiting evaluation of these perioperative factors. Although HgbA1c values were available for a subset of patients, they were insufficient for inclusion in multivariable models. Nevertheless, adjustment for BMI and the ECI offers a partial proxy for underlying metabolic burden.

Although Firth logistic regression was used to mitigate small-sample bias, some residual bias may persist. Medical and implant complications were aggregated into a single outcome, limiting granularity. Owing to its low incidence, regression analysis was not done for SSI, and these rare outcomes should be interpreted cautiously.

Regarding statistical power, we did not conduct post hoc power analyses, which are generally discouraged in the statistical literature because of their redundancy with observed *P*-values and potential to mislead.^39,40^ Power calculations are best done before study planning.^41^ Given that this was a retrospective observational study, our sample size was determined by the number of eligible patients available, making power planning inapplicable. We emphasized effect sizes and confidence intervals to ensure valid interpretation rather than relying solely on statistical significance.

### Future Research

Future research should focus on prospective or randomized trials assessing the effect of GLP-1RAs on both short-term and long-term outcomes after hip fracture surgery. Incorporating standardized measures of frailty, inflammatory biomarkers, and more granular glycemic control metrics could help clarify the mechanisms by which GLP-1RAs might influence surgical recovery. More extensive, multicenter studies with sufficient power to evaluate medical and surgical complications, functional recovery, and quality of life would further elucidate the role of GLP-1RAs in orthopaedic populations.

## Summary

T2DM patients on a GLP-1RA at the time of HA for FNF were not found to have a statistically significant increased risk for medical complications, IRs or EDEs, longer LOS, or 1-year implant failure; revision surgery; or mortality. ECI remains associated with an increased risk of poor outcomes after HA.

## Supplementary Material

SUPPLEMENTARY MATERIAL
