# Supplementary material for: Effect of Glucagon-like Peptide-1 Receptor Agonists on Outcomes After Hip Hemiarthroplasty for Femoral Neck Fractures in Patients With Type 2 Diabetes
Source: J Am Acad Orthop Surg Glob Res Rev. 2025 Oct 17;9(10):e25.00312. doi: 10.5435/JAAOSGlobal-D-25-00312 (PMC12543238; doi:10.5435/JAAOSGlobal-D-25-00312)
Supplement: SUPPLEMENTARY MATERIAL [file jagrr-9-e25.00312-s001.docx]

**Supplemental Digital Content**

Table 1: Logistic Regression Analysis of Medical and Implant Complications for Each Variable with Each Other Variable Controlled For

|  |  |  | ***30 Day Outcomes*** | | | ***1 Year Outcomes*** | | |
| --- | --- | --- | --- | --- | --- | --- | --- | --- |
|  |  | ***Length of Stay (d) (IRR)**** | ***Medical Complications*** | ***All-Cause Readmissions*** | ***Mortality/Discharge to Hospice*** | ***Implant Failure*** | ***Revision Surgery*** | ***Mortalit/Discharge to Hospice*** |
| GLP-1RA vs.  No GLP1RA | *OR* | 0.909 | 0.907 | 1.045 | 0.827 | 0.605 | 0.825 | 0.693 |
|  | *95% CI* | 0.825 to 1.002 | 0.566  to 1.453 | 0.710  to 1.538 | 0.343 to 1.996 | 0.291 to 1.256 | 0.347 to 1.959 | 0.384 to 1.248 |
|  | *P*^b^ | 0.0538 | 0.6844 | 0.8240 | 0.6727 | 0.1776 | 0.6621 | 0.2216 |
| Male vs.  Female | *OR* | 1.102 | 0.981 | 0.826 | 2.234 | 0.484 | 0.401 | 1.424 |
|  | *95% CI* | 1.003 to 1.212 | 0.620  to 1.555 | 0.564  to 1.211 | 0.988 to 5.049 | 0.222 to 1.053 | 0.149 to 1.077 | 0.818 to 2.479 |
|  | *P*^b^ | **0.044** | 0.9364 | 0.3279 | 0.0534 | 0.0674 | 0.0698 | 0.2115 |
| Age | *OR* | 0.998 | 0.980 | 0.977 | 1.023 | 0.971 | 0.998 | 1.036 |
|  | *95% CI* | 0.992 to 1.003 | 0.953  to 1.008 | 0.955  to 1.000 | 0.972 to 1.076 | 0.933 to 1.012 | 0.949 to 1.05 | 1 to 1.074 |
|  | *P*^b^ | 0.3889 | 0.1536 | 0.0504 | 0.3908 | 0.1604 | 0.9497 | 0.0517 |
| Smoker vs.  Nonsmoker | *OR* | 0.852 | 0.604 | 0.733 | 1.019 | 0.629 | 1.924 | 0.919 |
|  | *95% CI* | 0.716 to 1.015 | 0.251  to 1.451 | 0.371  to 1.452 | 0.213 to 4.87 | 0.16 to 2.465 | 0.56 to 6.614 | 0.306 to 2.761 |
|  | *P*^b^ | 0.0723 | 0.2593 | 0.3736 | 0.9811 | 0.5054 | 0.299 | 0.8802 |
| ECI | *OR* | 1.082 | 1.372 | 1.208 | 1.699 | 1.168 | 1.11 | 1.495 |
|  | *95% CI* | 1.056 to 1.108 | 1.219  to 1.545 | 1.093  to 1.336 | 1.38 to 2.093 | 0.986 to 1.385 | 0.905 to 1.36 | 1.289 to 1.734 |
|  | *P*^b^ | **<.0001** | **<.0001** | **0.0002** | **<.0001** | 0.0731 | 0.3161 | **<.0001** |
| Class 1 Obesity vs. Normal | *OR* | 0.974 | 0.991 | 1.034 | 1.289 | 1.246 | 1.329 | 0.717 |
|  | *95% CI* | 0.842 to 1.127 | 0.489  to 2.006 | 0.585  to 1.827 | 0.404 to 4.11 | 0.412 to 3.765 | 0.323 to 5.467 | 0.306 to 1.681 |
|  | *P*^b^ | 0.7228 | 2.006 | 0.8406 | 0.668 | 0.6966 | 0.6935 | 0.4446 |
| Class 2 Obesity vs. Normal | *OR* | 1.102 | 0.831 | 0.644 | 0.412 | 1.273 | 2.542 | 0.141 |
|  | *95% CI* | 0.887 to 1.368 | 0.284  to 2.432 | 0.263  to 1.574 | 0.044 to 3.857 | 0.261 to 6.208 | 0.477 to 13.556 | 0.016 to 1.266 |
|  | *P*^b^ | 0.3813 | 0.7936 | 0.3341 | 0.4371 | 0.7656 | 0.2746 | 0.0802 |
| Class 3 Obesity vs. Normal | *OR* | 1.528 | 0.490 | 0.612 | 1.16 | 2.714 | 2.312 | 1.984 |
|  | *95% CI* | 1.209 to 1.932 | 0.122  to 1.965 | 0.215  to 1.746 | 0.158 to 8.53 | 0.622 to 11.847 | 0.317 to 16.842 | 0.583 to 6.748 |
|  | *P*^b^ | **0.0004** | 0.3144 | 0.3589 | 0.8841 | 0.1842 | 0.408 | 0.2726 |
| Overweight vs.  Normal | *OR* | 1.049 | 1.220 | 0.880 | 0.74 | 1.303 | 1.661 | 0.819 |
|  | *95% CI* | 0.932 to 1.179 | 0.692  to 2.149 | 0.554  to 1.399 | 0.259 to 2.114 | 0.525 to 3.233 | 0.551 to 5.008 | 0.422 to 1.59 |
|  | *P*^b^ | 0.4283 | 0.4922 | 0.59 | 0.574 | 0.5681 | 0.3671 | 0.5548 |
| Underweight vs.  Normal | *OR* | 1.222 | 0.920 | 1.088 | 2.042 | 0.375 | 0.778 | 0.748 |
|  | *95% CI* | 0.944 to 1.581 | 0.234  to 3.623 | 0.378  to 3.131 | 0.279 to 14.97 | 0.02 to 7.139 | 0.04 to 15.106 | 0.145 to 3.856 |
|  | *P*^b^ | 0.1284 | 0.9055 | 0.8764 | 0.4823 | 0.5143 | 0.8681 | 0.7282 |

95% CI = 95% confidence interval, LOS = length of stay, OR = odds ratio, IRR,

^a^Negative binomial regression of the log count of initial hospital length of stay.

^b^ Values in bold indicate statistical significance (*P*< 0.05).
